# Incidence of Long-Term Pulmonary Vein Reconnection after a 2-Minute Cryoballoon Freeze for Pulmonary Vein Isolation—Invasive Insights of TTI-Dependent Cryoenergy Titration

**DOI:** 10.3390/jcdd9090284

**Published:** 2022-08-23

**Authors:** Alexander Pott, Michael Baumhardt, Mohammad Al-Masalmeh, Alexander Wolf, Matthias Schiele, Christiane Schweizer, Carlo Bothner, Deniz Aktolga, Yannick Teumer, Karolina Weinmann, Wolfgang Rottbauer, Tillman Dahme

**Affiliations:** Department of Medicine II, Ulm University Medical Center, 89073 Ulm, Germany

**Keywords:** atrial fibrillation, cryoballoon PVI, PV reconnection, time-to-isolation, freeze duration

## Abstract

Introduction: The optimal freeze duration in cryoballoon pulmonary vein isolation (PVI) is unknown. TTI-based titration of cryoenergy allows individualized freeze duration and has emerged as a favorable ablation strategy in PV cryoablation. In a recent study, we demonstrated that omission of a bonus freeze and reduction in freeze duration to a minimum of 2 min in the case of short TTI led to comparable arrhythmia recurrence rates. Whereas clinical outcome seems to be comparable to fixed freeze duration, evidence of long-term PV reconnection rates in patients undergoing TTI-based cryoballoon ablation is sparse. Aim of the study: To evaluate the procedural efficacy of a single 2-min freeze for PVI, we assessed PV conduction recovery after cryoballoon PVI with a TTI-guided titration of freeze duration compared to a fixed ablation protocol. Methods and Results: We included consecutive patients with atrial fibrillation (AF) recurrence undergoing a second ablation procedure after the initial cryoballoon procedure. The second AF ablation procedure was performed by the 3D-mapping system and radiofrequency ablation technique. A total of 219 patients (age: 66.2 ± 10.8 years, 53% female, paroxysmal AF: 53%) treated with the TTI-guided protocol (174 patients, 685 PV) or fixed protocol (45 patients, 179 PV) showed comparable total reconnection rates (TTI: 36.9% vs. fixed: 31.8%, *p* = 0.21). The PV reconnection rate was not statistically different for PVs treated with a 2-min freeze in case of short TTI, compared to longer freeze duration. Interestingly, the PV reconnection rate was lower in LIPVs treated with the fixed protocol (13% vs. 31%, *p* = 0.029). In the TTI group, 17 out of 127 patients (15%) had durable isolation of all PVs, whereas in 8 out of 40 patients (20%) in the fixed group, all PVs were still isolated (*p* = 0.31). Conclusions: overall reconnection rate was not different using a TTI-guided ablation protocol compared to a fixed ablation protocol, whereas the LIPV reconnection rate was significantly lower in patients treated with a fixed ablation protocol.

## 1. Introduction

Cryoballoon-based ablation for pulmonary vein isolation (PVI) in patients with either paroxysmal or persistent atrial fibrillation (parAF or perAF) is highly effective, as well as safe and is recommended as a first line therapeutic option [1]. Despite broad evidence that underlines the clinical benefit of cryoballoon-based PVI [2,3,4,5], the optimal freeze duration in cryoballoon (CB) PVI remains unknown. As recommended by medical device manufacturers and proven by several studies, initial cryoenergy application protocols provided at least two freeze cycles with 4 min freeze duration for every freeze cycle [6,7,8]. However, with the introduction of the second cryoballoon generation (CB2), it has been demonstrated that shorter cryoenergy application by reduction in freeze cycle duration, as well as omission of bonus freezes, led to comparable clinical outcomes [9,10,11].

In which type of patient and to which extent a reduction in cryoenergy application is useful remains under debate. In the last few years, several ablation protocols that take the time from the beginning of cryoenergy application to PV isolation (time-to-isolation, TTI) into account have been published [12,13,14].

TTI-based titration of cryoenergy allows individualized freeze duration and leads to reduced procedure duration, as well as reduced fluoroscopy time, whereas procedural efficacy remains stable. In a recent study, we demonstrated that omission of a bonus freeze and reduction in freeze duration to a minimum of 2 min in case of short TTI led to similar arrhythmia recurrence rates, compared to a non-TTI-based ablation protocol with at least two 4-min freezes per vein [14]. While the clinical outcome in patients that were treated with our TTI-based ablation protocol seems to be comparable to the fixed freeze duration, data on long-term PV reconnection rates after cryoballoon PVI are sparse [15,16]. Moreover, no remapping data of PV reconnection after a 2-min freeze compared to a 3-min freeze and to a 2 × 4-min freeze duration have been reported so far. The PV anatomy has an influence on the ablation procedure and PV isolation rates. However, the goal of our study was not to determine the influence of the PV anatomy but to analyze the effect of the freeze duration.

We aimed to investigate PVI durability of TTI-based ablation compared to fixed ablation duration in patients undergoing a repeat procedure due to atrial arrhythmia recurrence after initial cryo-PVI.

## 2. Methods

### 2.1. Study Population

**Figure 1:**
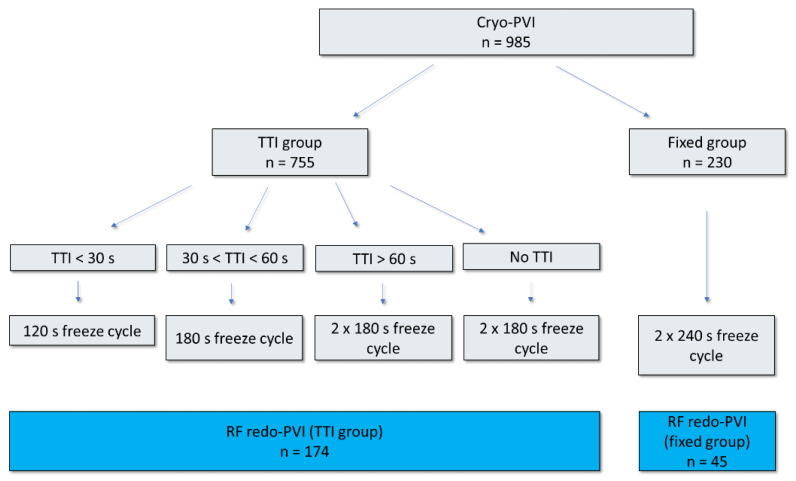
Flowchart that illustrates cryoenergy dosing that depends on TTI (TTI protocol) or a fixed ablation protocol (fixed protocol). Freeze cycles in the TTI protocol were adjusted depending on TTI. Subsequent RF redo procedures were performed in 174 patients (TTI group) and 45 patients (fixed group). PVI—pulmonary vein isolation; RF—radiofrequency ablation; TTI—time-to-isolation.

Consecutive patients with AF recurrence who underwent a second AF ablation procedure after initial cryoballoon PVI at Ulm University Medical Center were included. Initial cryoablation was performed either by a fixed protocol or a TTI-guided protocol (Figure 1). Written informed consent was obtained from each patient prior to the procedure and the protocol was approved by our local Ethics Committee. The investigation conformed with the principles outlined in the Declaration of Helsinki. The exclusion criteria were LA diameter of >60 mm, uncontrolled heart failure (NYHA class IV) and severe valvular disease.

### 2.2. Pre-Procedural Management

Intracardiac thrombi were ruled out by transesophageal echocardiography (TEE) prior to PVI. We did not perform PV computed tomography before the ablation. Oral anticoagulation with vitamin-K antagonists was not interrupted with a target INR of 2.0–3.0. Non-vitamin K antagonists (NOACs) were paused within 24 h prior to the procedure.

### 2.3. Initial Cryoballoon Pulmonary Vein Isolation Procedures

Cryoballoon PVI was performed as described before in detail [14]. A 12 F steerable sheath (Flexcath advance, Medtronic, Minneapolis, MN, USA) was positioned in the left atrium (LA) by a single transseptal puncture, followed by PV angiography. A 28 mm cryoballoon (Arctic Front Advance and Arctic Front Advance ST, Medtronic, Minneapolis, MN, USA) was advanced to the LA and guided to the target PV over a 10–25-mm spiral mapping catheter (Achieve or Achieve Advance, Medtronic, Minneapolis, MN, USA). In addition, 45 patients were treated with a fixed protocol with application of one or more freeze cycles of 240 s at each PV and a bonus freeze for each PV after documented PVI by performing entry- and exit-block testing. A total of 174 patients were treated with a time-to-isolation (TTI) guided protocol; the single freeze duration was adapted by the observed TTI. The freeze duration was shortened to 120 s by a TTI of <30 s. A TTI of <60 s resulted in a freeze duration of 180 s without a bonus freeze; a TTI of ≥60 s resulted in a single freeze of 180 s and a bonus freeze of 180 s. If PVI was not achieved following a single freeze application, an additional freeze was administrated until electrical isolation was verified.

### 2.4. Radiofrequency Pulmonary Vein Isolation Procedure

In all patients, repeat ablation was performed by using irrigated-tip radiofrequency ablation in combination with a 3D-mapping system (Carto3, Biosense Webster, Irvine, CA, USA or NavX Ensite Velocity, St. Jude Medical, Saint Paul, MN, USA). A 10-polar diagnostic catheter was placed in the coronary sinus (CS). Subsequently, a circular mapping catheter (LASSO^®^ 2515 Variable Mapping Catheter, Biosense Webster, Irvine, CA, USA or Inquiry AFII™ Circular Mapping Catheter, 15 or 20 mm, St. Jude Medical, Saint Paul, MN, USA) was advanced to the left atrium via a 12 F steerable sheath (8.5 F, Agilis NXT; St. Jude Medical, Saint Paul, MN, USA) and an ablation catheter with or without contact-force sensing (Smarttouch SF, Biosense Webster, Irvine, CA, USA or TactiCath or CoolFlex, St. Jude Medical, Saint Paul, MN, USA) was delivered to the left atrium via a non-steerable sheath (SL1, St. Jude Medical, Saint Paul, MN, USA) after double transseptal puncture. RF ablation power was between 25 and 35 W, temperature limit was at 43 °C, and saline irrigation rate at 17–30 mL/min. During ablation at the posterior wall and in the CS, RF power was restricted to 25 W.

Left atrial voltage-mapping for the detection of low-voltage areas (LVA) in sinus rhythm was performed in every redo patient (voltage borders: 0.2–0.5 mV). The extent of LVA in the left atrium was quantified as follows: degree I demonstrated < 25% LVA, degree II 25–50% LVA, degree III >50% LVA and degree IV >75% LVA.

### 2.5. Statistical Analysis

Statistical analyses were performed using SPSS^®^ software (SPSS, V27, Chicago, IL, USA). Categorical variables are described as absolute and relative frequencies and continuous variables are expressed as mean ± SD. Categorical variables were compared using a Fisher’s exact test or chi-square test and continuous variables that were normally distributed were compared by a two-tailed *t*-test, otherwise the Mann–Whitney test was performed. Event-free survival was estimated using Kaplan–Meier analysis and statistically compared by the log-rank test. A *p*-value < 0.05 was considered statistically significant.

## 3. Results

### 3.1. Baseline Characteristics

Out of the 985 AF patients treated with the cryoballoon procedure between 2013 and 2020 at Ulm University Medical Center, we included 219 patients with recurrence of atrial tachycardia (AT) and/or AF undergoing a second AF ablation procedure. Whereas 45 (21%) patients were initially treated with a fixed cryoenergy dosing protocol (fixed group), 174 (79%) patients had been treated during the first ablation procedure using a TTI-based ablation strategy (TTI group, Figure 1).

The mean age of the patients was 66.8 ± 10.5 years, 108 out of 219 (49.3%) patients were female, and 103 out of 219 (47.0%) patients had perAF. Mean left atrial diameter (LAD) was 45.8 ± 6.3 mm and mean CHA_2_DS_2_-VaSc-Score was 3.2 ± 1. Baseline characteristics between the TTI group and fixed group were not different; however, there was a trend towards the proportion of patients with persistent AF being higher in the TTI group (TTI: 88/174 (51%) vs. fixed: 15/45 (33%), *p* = 0.06). In addition, the mean age of patients and proportion of female patients who underwent a second AF ablation procedure were numerically higher in the TTI group (Table 1).

### 3.2. Procedural Data

During the initial cryoballoon PVI, the mean cumulative number of freezes was 9.0 ± 1.6 in the fixed group vs. 6.4 ± 2.2 (*p* < 0.001) in the TTI group, with a mean total freeze duration of 34.6 ± 6.5 min in the fixed group, compared to 17.0 ± 6.2 min (*p* < 0.001) in the TTI group. RF procedure duration for redo procedures was 175.2 ± 61.0 min in the fixed group and 161.5 ± 56.5 min in the TTI group (*p* = 0.22). In addition, the number and duration of applied RF lesions did not differ between either study group (Table 2).

In the fixed group, 179 PVs in 45 patients were identified with 2 out of 45 (4.4%) patients with a left common pulmonary vein (LCPV) and 1 out of 45 patients (2.2%) with a left middle pulmonary vein (LMPV). In the TTI group, 685 PVs in 174 patients were identified with 12 out of 174 patients (6.9%) with a LCPV and 1 out of 174 (0.6%) patients with a right middle pulmonary vein (RMPV). Interestingly, despite the higher proportion of perAF patients in the TTI group, the extent of LVA in the left atrium did not differ significantly between either study group. Incidence of procedure-associated complications were not different between the TTI and the fixed group (Table S1).

### 3.3. PV Reconnection Rate after Initial Cryoballoon PVI

Overall, 310 out of 864 (36%) PVs were classified as reconnected during the redo ablation procedure, with the highest reconnection rate for LCPV (10/14 (71%)) and lowest reconnection rate for the LIPV (52/205 (25%)). In the fixed group, 57 out of 179 (32%) PVs were classified as reconnected compared to 253 out of 685 (37%) reconnected PVs in the TTI group (*p* = 0.21).

**Figure 2:**
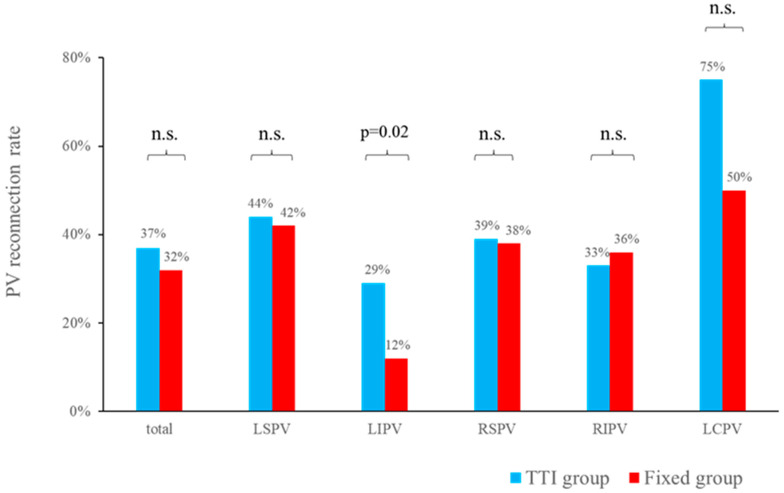
Total and per vein incidence of PV reconnection in the TTI group and fixed group, showing no significant difference in total or LSPV, RSPV, RIPV and LCPV reconnection rate, but significantly lower LIPV reconnection rate in the fixed group.

The reconnection rate per PV was also not different for LSPV, RSPV and RIPV between either study group. However, in the fixed group, the rate of LIPV reconnection was significantly lower in comparison to the TTI group (TTI: 47/167 (29%) vs. fixed: 5/43 (12%); *p* = 0.02, Table 2, Figure 2).

**Figure 3:**
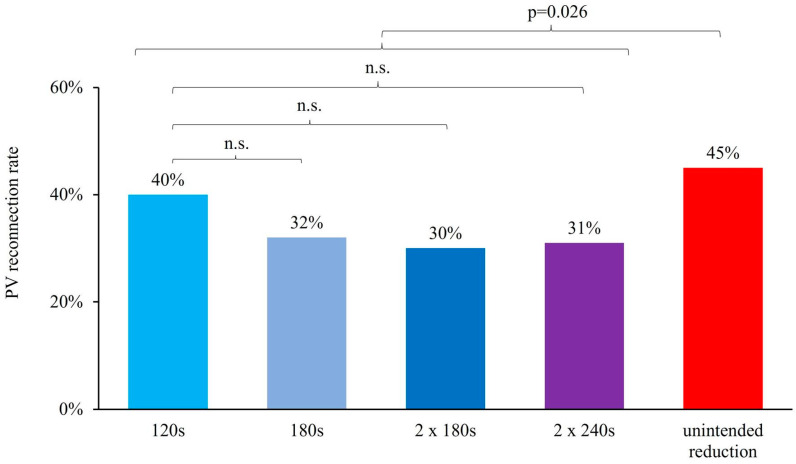
PV reconnection rate that depends on freeze duration during initial cryo-PVI shows no significant difference in PVs treated with intended freeze duration but significantly higher PV reconnection rates in unintended reduction in freeze duration (loss of phrenic nerve activity, e.g.).

According to the applied freeze duration during initial cryo-PVI, we analyzed the PV reconnection rates for PVs, which were initially isolated with 120 s, 180 s, 2 × 180 s. (TTI group) and 2 × 240 s. (fixed group). Interestingly, we found that the PV reconnection rate for PVs initially treated by a 120 s freeze duration was not statistically higher compared to the longer freeze duration in either the TTI group or the fixed group (Figure 3).

Interestingly, the highest PV reconnection rate (45%) was found for PVs, in which applied freeze duration was shorter than intended freeze duration (mostly caused by premature abortion of cryoenergy application due to safety reasons) and the lowest PV reconnection rate (30%) was found for PVs, in which freeze duration was 2 × 180 s, according to the TTI ablation protocol. By analyzing the PV reconnection rate for PVs, in which the intended freeze duration was equal to the effective freeze duration in comparison to the unintended reduction in freeze duration, we found that the reconnection rate was significantly lower (intended: 200/605 PV (33%) vs. unintended 100/223 PV (45%), *p* = 0.026).

### 3.4. Localization of PV Conduction Recovery

**Figure 4:**
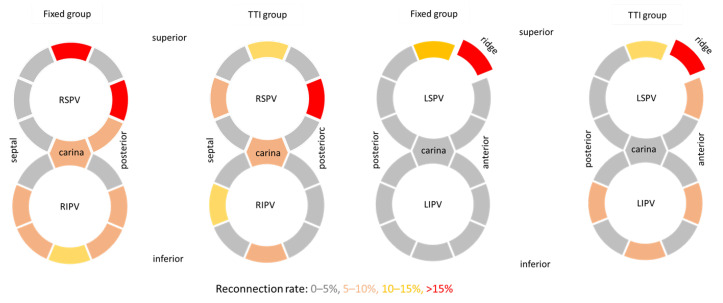
Segmental localization of electrical reconduction gaps at the PV ostium during RF redo procedure. Colors indicate the number of reconnections found in each segment, showing predominant LSPV reconduction gaps at the ridge and RSPV reconduction gaps (anterior and superior).

In addition to the PV reconnection rate, we analyzed the localization of PV reconnection for PVs treated by our TTI-based cryo-PVI in comparison to the fixed cryoenergy application protocol, allocating every reconnection gap to one of the eight segments at PV ostium (Figure 4). Although there was no statistically significant difference in PV reconnection location, PV reconnections of the LSPV were predominantly located at the ridge, whereas RSPV reconnection hotspots were superior and anterior for both the fixed and TTI group. In the case of RIPV reconnection, gaps were found typically at the inferior part of the RIPV ostium. Numerically, at the septal carina, reconduction gaps were found more often compared to the lateral carina.

## 4. Discussion

Despite the high procedural success rate during PVI for AF treatment, a relevant number of patients experience AF recurrence during further clinical follow-ups. Irrespective of the applied ablation energy, cryoenergy or radiofrequency (RF) energy, during index procedures, PV reconnection in AF patients undergoing a redo ablation procedure are found regularly [14,15,17].

TTI-based cryoenergy application has emerged as the method of choice in AF patients treated with the cryoballoon procedure.

To the best of our knowledge, our TTI-based ablation protocol is the only ablation approach that allows the reduction in cryoenergy application to a single two-minute freeze if TTI is <30 s, leading to a tremendous reduction in procedural duration, mainly due to reduction in LA dwell time, and fluoroscopy time [14]. Whether long-term PV reconnection rates in patients that were treated by our TTI-based cryoenergy ablation protocol differ from PV reconnection rates in patients treated with a fixed ablation protocol is unknown. Hence, in the present study, we analyzed PV reconnection rates in AF recurrence patients after initial cryo-PVI, with either TTI-based or fixed cryoenergy application, that were referred for redo ablation procedures.

To date, our study offers the largest number of patients after initial cryo-PVI undergoing a redo ablation procedure and provides interesting invasive insights about the incidence and characteristics of PV conduction recovery in AF recurrence patients. The major findings are as follows: (1) PV reconnection in the TTI group (37%) was not statistically different compared to the fixed group (32%). (2) A single two-minute freeze in case of TTI < 30 s does not lead to higher recurrence rates. (3) The highest PV reconnection rate was found after unintended reduction in freeze duration due to safety reasons, such as phrenic nerve palsy (PNP) or low esophageal temperature. (4) The LIPV reconnection rate was significantly higher in the TTI group compared to the fixed group.

### 4.1. Total PV Reconnection Rate

In a FIRE and ICE subgroup analysis, the PV reconnection rate in AF recurrence patients after initial RF-PVI was nearly 50% and approximately 35% after initial cryo-PVI [17].

Lower reconnection rates after non-TTI cryo-PVI have been reported by Aryana and co-workers. Interestingly, the reported PV reconnection rates after RF-PVI were also lower compared to the above-mentioned FIRE and ICE subgroup analysis [18].

Higher PV reconnection rates have also been reported in a small study by Wieczorek et al. who compared PV reconnection patterns in AF recurrence patients after initial phased multielectrode radiofrequency technology (PVAC)-PVI versus cryo-PVI, with a fixed ablation approach of 2 × 240 s per PV. Remarkably, the PV reconnection rate in the the PVAC group reported in this study was 75% and 52% in the cryo-PVI group [19].

Data on PV reconnection rate after a TTI-guided ablation protocol vs. a fixed cryoenergy application have been published recently. Interestingly, Chen et al. demonstrated similar PV reconnections rates in patients treated by a TTI-guided protocol compared to our findings, whereas fixed cryoenergy application was found to result in significantly lower PV conduction recovery [16].

Taken together, the broad variety in the evaluation of PV isolation status reported in several redo ablation studies [15,20,21,22] indicates that accurate determination of PV isolation status is still challenging. For instance, operator-dependent factors, such as technical skills and in-depth knowledge of electrophysiology, may influence PV reconnection detection, as well as institutional-specific aspects, such as standard operating procedures, drug application or the mapping system used.

In our study, with a total PV reconnection rate of 36%, our incidence of PV conduction recovery is in the same range as the FIRE and ICE subgroup analysis and other studies from high-volume ablation centers. This suggests that our findings might also be influenced by operator- or institutional-specific bias in the determination of PV reconnection status, but does not seem to lead to an under- or overestimation of PV conduction recovery in our study.

### 4.2. Influence of Freeze Duration and Type of PV for PV Reconnection

Induced myocardial damage by energy application for PVI strongly depends on the type and duration of the applied energy [23]. More intensive cryoenergy applications have been associated with higher incidences of safety endpoints, such as PNP or low oesophageal temperatures during cryo-PVI, whereas less intensive approaches show excellent clinical outcomes, with a significantly reduced incidence of PNP and reductions in oesophageal temperature [24]. However, the optimal freeze duration regarding clinical efficacy, as well as procedural safety, is still unknown.

Hence, the aim of our study was to assess PV reconnection rates in AF recurrence patients after a single 2-min freeze in the case of quick PV isolation (TTI < 30 s) during initial ablation procedure. Remarkably, we found that the PV reconnection rate was not statistically different compared to PV initially isolated by a more intensive application of cryoenergy. These data indicate that (1) TTI is an adequate patient-specific biomarker for energy titration and (2) that a single 2-min freeze is not too short for durable PV isolation in the case of quick PV isolation.

However, two points must be considered in the evaluation of our TTI-based cryoenergy protocol. First, despite finding no statistical difference in PV reconnection, numerically, PV reconnection was higher for a single 2-min freeze compared to longer TTI-based and non-TTI based freeze durations. Whether this numerical difference might become statistically significant by enlarging our study population cannot be excluded. However, even if further studies demonstrate higher PV reconnection rates for PVs treated with a single 2-min freeze duration, this should not automatically imply higher AF recurrence rates.

Second, the LIPV reconnection rate in the fixed group was significantly lower compared to the TTI group, suggesting that TTI-based titration of cryoenergy application leads to an unfavorable long-term procedural result. However, the number of reassessed LIPVs in the fixed group is rather small. Hence, whether this statistical difference is a chance finding or indicates a true mismatch of cryoenergy delivery for durable LIPV isolation cannot be ultimately answered by our study.

As a consequence of our findings, we suggest considering the extension of cryoenergy application by either increasing freeze duration or by application of a bonus freeze for LIPV isolation in the case of insufficient further procedural parameters, such as incomplete PV occlusion or insufficient decline in balloon temperature.

## Figures and Tables

**Table 1:** Baseline characteristics.

Baseline Characteristics	Total (n = 219)	Fixed Group (n = 45)	TTI Group (n = 174)	*p*-Value
Age [y] [mean ± SD]	66.8 ± 10.5	64.8 ± 11.6	67.3 ± 10.2	0.16
Sex (female) [n (%)]	108 (49%)	18 (40%)	90 (52%)	0.16
Pers. AF [n (%)]	103 (47%)	15 (33%)	88 (51%)	0.06
BMI [kg/m^2^] [mean ± SD]	28.8 ± 5.5	29.1 ± 5.4	28.7 ± 5.5	0.67
LAD [mm] [mean ± SD]	45.8 ± 6.3	45.1 ± 6.7	46.0 ± 6.2	0.44
CHA^2^DS^2^-VASc [mean ± SD]	3.2 ± 1.6	3.0 ± 1.6	3.2 ± 1.6	0.61
Congestive heart failure [n (%)]	58 (27%)	9 (20%)	49 (28%)	0.27
Hypertension [n (%)]	177 (81%)	36 (80%)	141 (81%)	0.88
Diabetes mellitus [n (%)]	37 (17%)	7 (16%)	30 (17%)	0.79
Stroke [n (%)]	20 (9%)	7 (16%)	13 (8%)	0.09
Vascular disease (%)	71 (32%)	14 (31%)	57 (33%)	0.83

Categorical variables are expressed as absolute and percentage (in parentheses). Continuous variables are expressed as mean ± SD. AF, atrial fibrillation; BMI, body mass index; LAD left atrial diameter.

**Table 2:** Procedural characteristics.

Procedural Parameters	Total (n = 219)	Fixed Group (n = 45)	TTI Group (n = 174)	*p*-Value
1st ablation (Cryo)				
Procedure duration [min] [mean ± SD]	96.6 ± 34.0	131.5 ± 28.3	87.5 ± 29.3	**<0.001**
Total freeze duration [min] [mean ± SD]	20.6 ± 9.5	34.6 ± 6.5	17.0 ± 6.2	**<0.001**
Number of freezes [mean ± SD]	6.9 ± 2.4	9.0 ± 1.6	6.4 ± 2.2	**<0.001**
2nd ablation (RF)				
Procedure duration [min] [mean ± SD]	164.4 ± 57.6	175.2 ± 61.0	161.5 ± 56.5	0.22
Total applied RF energy [min] [mean ± SD]	17.1 ± 16.6	20.9 ± 19.9	16.1 ± 15.6	0.14
Number RF applications [mean ± SD]	20.3 ± 41.2	19.2 ± 38.7	20.6 ± 41.9	0.85
Total PV reconnection 2nd ablation:	310/864 (36%)	57/179 (32%)	253/685 (37%)	0.21
LSPV	90/205 (44%)	18/43 (42%)	72/162 (44%)	0.76
LIPV	52/205 (25%)	5/43 (12%)	47/162 (29%)	**0.02**
LCPV	10/14 (71%)	1/2 (50%)	9/12 (75%)	0.51
RSPV	85/219 (39%)	17/45 (38%)	68/174 (39%)	0.87
RIPV	73/219 (33 %)	16/45 (36%)	57/174 (33%)	0.72
Mean degree of low voltage area [mean ± SD]	1.8 ± 1.2	2.2 ± 1.2	1.8 ± 1.2	0.14

## Data Availability

The datasets generated during and/or analysed during the current study are available from the corresponding author on reasonable request.

## Supplementary Materials

The following supporting information can be downloaded at: https://www.mdpi.com/article/10.3390/jcdd9090284/s1, Table S1: Complication rates during ablation procedures.
