# Supplementary material for: Incidence of Long-Term Pulmonary Vein Reconnection after a 2-Minute Cryoballoon Freeze for Pulmonary Vein Isolation—Invasive Insights of TTI-Dependent Cryoenergy Titration
Source: J Cardiovasc Dev Dis. 2022 Aug 23;9(9):284. doi: 10.3390/jcdd9090284 (PMC9505807; doi:10.3390/jcdd9090284)
Supplement: Supplementary file 1 [file jcdd-09-00284-s001.zip › jcdd-1737031-supplementary.pdf]

**Procedural complications**	**Total (n= 219)**	**Fixed group (n= 45)**	**TTI group (n= 174)**	**p-value**
Total	15 (6,8%)	4 (8,9%)	11 (6,3%)	0.54
1st ablation (Cryo)	9 (4,1 %)	1 (2,2%)	8 (4,6%)	0.47
Pericardial effusion/tamponade	2 (0,9%)	0	2 (1,2%)	n/a
Phrenic nerve palsy	5 (2,3%)	1 (2,2%)	4 (2,3%)	0.98
Vascular access complication	2 (0,9%)	0	2 (1,2%)	n/a
PV stenosis	0	0	0	n/a
Atrioesophageal fistula	0	0	0	n/a
Esophageal lesions	0	0	0	n/a
Stroke/Transient ischemic attack	0	0	0	n/a
Hemodynamic instability	0	0	0	n/a
Death	0	0	0	n/a
2nd ablation (RF)	6 (2,7%)	3 (6,7%)	3 (1,7%)	0.07
Pericardial effusion/tamponade	4 (1,8%)	2 (4,4%)	2 (1,2%)	0.14
Phrenic nerve palsy	0	0	0	n/a
Vascular access complication	1 (0,5%)	0	1 (0,6%)	n/a
PV stenosis	0	0	0	n/a
Atrioesophageal fistula	0	0	0	n/a
Esophageal lesions	0	0	0	n/a
Stroke/Transient ischemic attack	0	0	0	n/a
Hemodynamic instability	1 (0,5%)	1 (2,2%)	0	n/a
Death	0	0	0	n/a
